# Is theatre utilization a valid performance indicator for NHS operating theatres?

**DOI:** 10.1186/1472-6963-8-28

**Published:** 2008-01-31

**Authors:** Omar Faiz, Paris Tekkis, Alistair Mcguire, Savvas Papagrigoriadis, John Rennie, Andrew Leather

**Affiliations:** 1 Department of Surgery, St Mark's Hospital, Harrow, Middlesex, HA1 3UJ, UK; 2 Imperial College London, Department of Surgical Oncology and Technology, St Mary's Hospital, Praed Street, London W21NY, UK; 3 Department of Health and Social Care, Cowdray House, London School of Economics and Political Science, Houghton Street WC2A 2AE, UK; 4 Department of General Surgery, Kings College Hospital, Denmark Hill, London SE5 9RS, UK

## Abstract

**Background:**

Utilization is used as the principal marker of theatre performance in the NHS. This study investigated its validity as: a managerial tool, an inter-Trust indicator of efficient theatre use and as a marker of service performance for surgeons.

**Methods:**

A multivariate linear regression model was constructed using theatre data comprising all elective general surgical operating lists performed at a NHS Teaching hospital over a seven-year period. The model investigated the influence of: operating list size, individual surgeons and anaesthetists, late-starts, overruns, session type and theatre suite on utilization (%).

**Results:**

7,283 inpatient and 8,314 day case operations were performed on 3,234 and 2,092 lists respectively. Multivariate analysis demonstrated that the strongest independent predictors of list utilization were the size of the operating list (p < 0.01) and whether the list overran (p < 0.01). Surgeons differed in their ability to influence utilization. Their overall influence upon utilization was however small.

**Conclusion:**

Theatre utilization broadly reflects the surgical volume successfully admitted and operated on elective lists. At extreme values it can expose administrative process failure within individual Trusts but probably lacks specificity for meaningful use as an inter-Trust theatre performance indicator. Unadjusted utilization rates fail to reflect the service performance of surgeons, as their ability to influence it is small.

## Background

Utilization has become the principal measure of NHS operating theatre service performance. In part, the current reliance on utilization has arisen from its historical use in foreign, often privatised, healthcare systems [1-5]. In addition however, major recent Audit Commission [6,7] and Modernisation Agency [8] publications have served to enhance the profile of this performance indicator in the United Kingdom.

Nearly seven million operations are performed each year in the NHS [9]. In the 2002/03 financial period the annual budget for main theatre departments in acute Trusts in England and Wales exceeded £1 billion [10]. As such, hospital theatres represent a significant expense. Efficient use of this costly resource is therefore economically desirable. In addition to financial reasoning – the current political pressures on waiting lists serve to amplify the importance of effecting efficient theatre usage. At present, approximately 1 million people are awaiting NHS treatment [11]. In order to achieve the governments aim to progressively shorten total waiting times to less than 18 weeks by 2008 [12] – enhanced theatre capacity is required. To this end service change has involved various government initiatives including: a promotion of day case operating [13-15] as well as the development of independent *Treatment Centres *[12,16]. In addition to these measures however, a requirement to increase efficiency amongst theatre units within acute NHS Trusts is also recognized.

Despite the widespread use of utilization rates in the public setting there has been little research to date investigating its validity as a performance indicator. The purpose of this study was to investigate the factors that influence elective general surgical theatre list utilization within an NHS hospital. As such, the study sought to assess the validity of utilization as a performance indicator that could be used to benchmark theatre performance between Trusts as well as a tool that could be used by individual Trusts to facilitate managerial decision-making. In addition, this investigation aimed to explore the influence of individual surgeons on utilization and thereby assess its potential use as a marker of their service performance.

## Methods

### Data methods

The study data comprised all elective day case (DC) and inpatient general surgical operations performed at a Teaching Hospital between April 1997 and April 2004. Prospectively entered data relating to the: procedure type, timings and personnel involved in operations were retrieved from the hospital theatre database (*Surgiserver ^© ^McKennon systems*). Operations were aggregated into operating lists. Procedure durations were calculated through subtraction of the recorded time when anaesthetic administration was commenced from the time of surgical drape removal at the end of the procedure. Database variables were consequently recoded into: list, session and personnel factors (see below). The latter, in addition to operating list size, represented the utilization covariates investigated in this study.

#### Study endpoint

Operating list utilization rates represented the principal study outcome measure. These were calculated through division of the sum of total list procedure time by the allocated session duration. Utilization rates were expressed as percentages.

#### Study covariates

database variables were recoded into: operating list size as well as session, personnel and list factors.

##### a) Calculation of "operating list size"

A scoring system was developed from all operative procedures to quantify the size of general surgical operating lists. This system that was developed we termed the *Operative Score of Complexity index*. It has been applied to the measurement of workload and productivity in inpatient and outpatient theatres separately *(In Press)*. Specifically, a numerical *case-score *(measured in units) was assigned to each *Office of Population Census and Statistics-4 *(OPCS-4) code on the basis of the historical median case duration of all operative procedures that had been assigned to the corresponding code. The actual numerical score represented the procedure median duration (in seconds)/30. The latter calculation was performed to simplify the numerical score to a tangible figure. For example, the *case-score *of a day surgery primary inguinal hernia repair was 106 units. This numerical value represented the median duration (in seconds)/30 of all procedures that had been performed in the day surgery department during the study period and coded to the 'Primary Repair of Inguinal Hernia' OPCS-4 code. *Case-score *indices were calculated separately for the main theatre (MT) and day surgery (DS) databases to account for differences in complexity between operations performed in the respective departments. The sum of the case-scores of constituent list procedures derived the output of individual operating lists (*i.e. list-scores*). In MT's an adjustment was made to *list-scores *(i.e. list-score/hour of allocated session time) in order to overcome heterogeneity of session duration. As 99.2% of all day surgery cases were performed on 4-hour operating lists, output was recorded as the list-score without adjustment.

##### b) Session factors

Operating lists were recoded according to whether they took place on 'morning', 'all-day' or 'afternoon' sessions. In addition, lists were classified according to the departmental theatre suites where surgery was undertaken (see Table 1).

**Table 1:** A summary of general surgical operating list characteristics in the DS and MT departments between 1997 & 2004.

**Operating list factors**	**Day Surgery (DS)**	**Main Theatres (MT)**
***Operating list volume***		
Mean list-score in units per hour (SD)	70.3(26.0)	86.86 (38.29)
***Session factors***		
Session type		
Percentage of operations performed on Morning lists (n)	38.2%(3226)	15.9%(1156)
Percentage of operations performed on Afternoon lists (n)	61.1%(5083)	17.4%(1265)
Percentage of operations performed on 'All-day' lists (n)	-	66.8%(4862)
No. of theatre suites	5	10
***Personnel factors***		
***Surgeons***		
Total number of surgeons coded on database	133	125
No. of surgeons with >100 operative procedures	16	16
Percentage of total cases performed by surgeons with>100 cases (n)	79.3% (6594)	78.7% (5732)
***Anaesthetists***		
Total no. of Anaesthetists' coded on database	246	238
No. of anaesthetists with >100 operative procedures	10	14
Percentage of total cases performed by anaesthetists with>100 cases (n)	23. 9% (1983)	65. 6%(5290)
***List factors***		
***Overruns***		
Overrunning operating lists (%)	627/2092 (30.0%)	1079/3234 (33.3%)
Median list overrun (Q1-Q3,n) in minutes	50 (24 – 84, n = 627)	-
Median list overrun (Q1-Q3,n) as a percentage of session duration (%)	-	13.1(5.5–26.2, n = 1079)
Number.(%) of MT lists where no overrun occurred	-	2262 (69.9%)
Number(%) of MT lists where overrun : session length = 0–0.1	-	403 (12.5%)
Number(%) of MT lists where overrun : session length = 0.11–0.2	-	215 (6.6%)
Number(%) of MT lists where overrun : session length = 0.21–0.3	-	150 (4.6%)
Number(%) of MT lists where overrun : session length >0.31	-	204 (6.3%)
***Late-starts***		
Median (Q1-Q3, n) late-start in minutes	32 (17–48, 2087)	65 (41–90, 3229)
%(n). DSC operations on lists where Late start <30 minutes	996 (47.61%)	-
%(n). DSC operations on lists where Late start is 30–60 minutes	870 (41.59%)	-
%(n). DSC operations on lists where Late start is > 60 minutes	221 (10.56%)	-
Number (%) of MT lists where no Late-start occurred	-	103 (3.18%)
Number(%) of MT lists where Late-start: session length = 0–0.1	-	730 (22.57%)
Number(%) of MT lists where Late-start : session length = 0.11–0.2	-	1564 (48.36%)
Number(%) of MT lists where Late-start : session length = 0.21–0.3	-	522 (16.14%)
Number(%) of MT lists where Late-start : session length > 0.31	-	315 (9.74%)

##### c) Personnel factors

Surgical and anaesthetic practitioners were included in day surgery and main theatre analyses on an anonymous individual basis if they had performed more than 100 operative procedures (see Table 1). Practitioners that had performed less than 100 cases were pooled into separate surgical and anaesthetic personnel categories respectively.

##### d) List factors

List factors describe the extent to which operating sessions started late or overran the allocated session time. An overrun was defined to have occurred when the last procedure on an operating list finished beyond the scheduled finish time. A binary approach to day surgery overruns (i.e. overrun, no-overrun) was used because even minor time infringements in this setting may have adverse staffing consequences. Late-starts in the day surgery setting were however categorised according to the time delay incurred. Overruns and late-starts in MT's were categorised according to the proportion of time infringement as a function of session length. The latter was necessary to compensate for varying session length in MT's. The specific definitions of late-start and overrun categories for the DS and MT data are described in Table 1.

### Statistical Analysis

Unifactorial regression analysis was used to identify risk factors related to theatre utilization. Utilization outcome measures assumed a normal distribution and no data transformation was required. Operating list size (i.e. list-score units) was entered into the models as a continuous variable as it demonstrated a clear linear relationship with utilization in both DS and MT models. Other independent risk factors (i.e. list, session and personnel factors) were entered into the models as categorical variables.

In order to determine the adjusted relationship between list utilization and other variables including: the size of the list, list factors, (overruns and late-starts), personnel and session factors (session type and theatre suite), multiple linear regression models were constructed for the DS and MT departments respectively by entering influential univariate risk factors. Stepwise regression was used to evaluate individual predictors. Criteria were set so that variables were excluded from model if their probability of influence was low (p > 0.1). The mean ± Standard Deviations (SD) and median (interquartile range, n) values were recorded for outcomes as appropriate. For all tests of significance, P < 0.05 was considered statistically significant.

## Results

### Operating list characteristics

Throughout the study period 7283 operations were performed on 3234 general surgical operating lists in the MT department. Over the same period 8314 operations were carried out on 2,092 lists in the DS centre. Nearly all (97.6%) patients that were operated in MT's were performed under general anaesthesia (GA) whereas in the DS centre 61.6%, 29.8% and 7.7% operations were performed under GA, 'Local Infiltration' and 'Sedation' respectively.

The descriptive characteristics of the operating lists performed in the DS and MT departments throughout the study period are described in Table 1. The sub-categories of list, session and personnel factors are described in accordance with the categories entered in the regression analyses.

### Theatre list utilization rates

**Figure 1:**
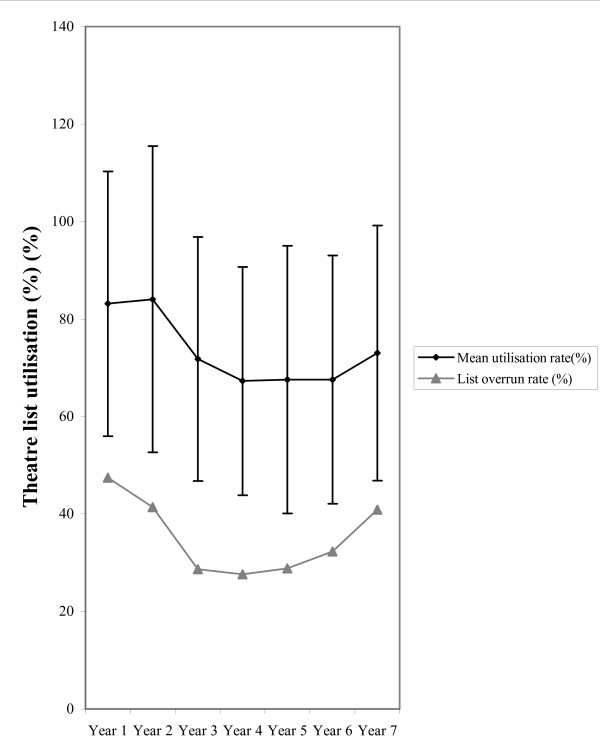
Mean utilization (+SD) and annual overrun rates for main theatre general surgical lists between 1997 & 2004.

**Figure 2:**
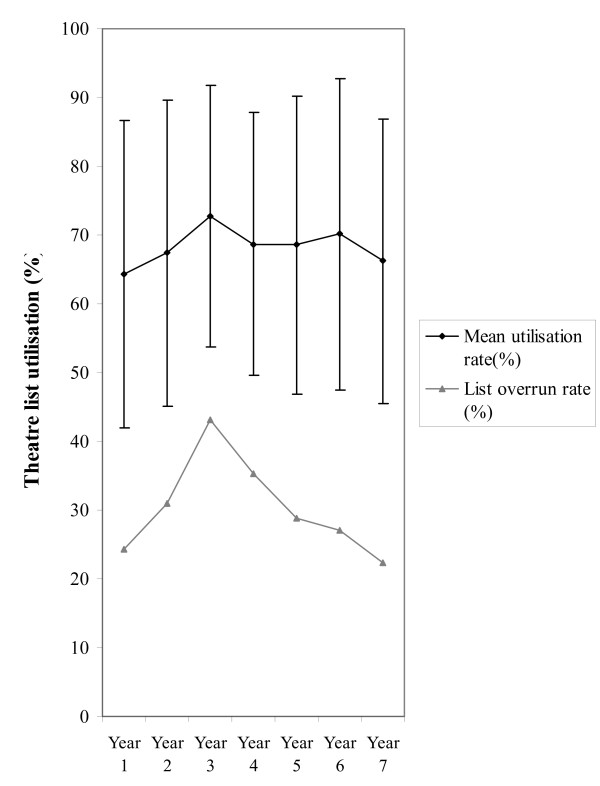
Mean utilization (+SD) and annual overrun rates for day theatre general surgical lists between 1997 & 2004.

Throughout the study period the mean theatre list utilization rate was 73.2% (SD: 27.5%, n = 3234) and 68.2% (SD: 21.4, n = 2087) in the MT and DS departments respectively. Over the same time period 30% (n = 627) of day surgery lists and 33% (n = 1079) of main theatre lists overran. Figures 1 &2 demonstrate the annual mean theatre utilization rates and the corresponding annual overrun rates. An association between utilization rates and overruns was observed in the MT and DS departments.

The results of the constructed regression models are shown in Tables 2 (DS) and 3 (MT). The latter tables can be used to predict list utilization rates by extrapolation from the regression equation y = a+b(x), where y = the predicted utilization rate, a = the model constant (or intercept) and b = the regression (Beta) coefficient of covariate(x). For example, a list utilization rate prediction can be made for a hypothetical scenario where a day surgery list with 300 list-score units are operated upon by surgeon 12 and all other session variables correspond to reference categories (i.e. the list does not overrun and starts promptly and is carried out by anaesthetist 1). The predicted utilization rate for this scenario equates to the model constant (38.1%) + (300 list-score units × Beta coefficient for list size i.e. 0.093)% + (1 × 3.8% i.e. the Beta coefficient for surgeon 12) + nil else (as all other covariates were the reference categories). Therefore the predicted utilization rate for this list scenario = 69.8%.

**Table 2:** Multiple regression model for Day Surgery list utilization. (only reference categories and retained model variables listed).

***Risk Factor***	***Model coefficient***	***Beta coefficent***	***Beta Standard Error***	***p-value***	***Change in R-Square***	***Model R-Square***
***Model Constant***	38.1	-	0.6	<0.01	-	0.603
***List volume (1 (unit)***		0.093	0.0	<0.01	0.394	
***List factors***						
***List Overruns***		19.2	0.4	<0.01	0.152	
***Late-starts***						
*<30 mins*		Ref				
*30–60 mins*		-3.1	0.3	<0.01	0.007	
*>1 hr*		-10.2	0.6	<0.01	0.014	
***Personnel factors***						
***Surgeon ***						
*Surgeon 1*		Ref				
*Surgeon 2*		-13.2	0.9	<0.01	0.004	
*Surgeon 3*		-3.8	0.5	<0.01	0.002	
*Surgeon 5*		-5.4	1.1	<0.01	0.001	
*Surgeon 7*		-8.7	0.6	<0.01	0.005	
*Surgeon 8*		-12.8	1.3	<0.01	0.004	
*Surgeon 9*		-7.3	1.2	<0.01	0.001	
*Surgeon 10*		-4.8	0.9	<0.01	0.001	
*Surgeon 11*		-7.4	0.6	<0.01	0.004	
*Surgeon 12*		3.8	0.7	<0.01	0.007	
*Surgeon 14*		-2.7	1.4	0.05	0.000	
*Surgeon 'others'*		-4.4	0.5	<0.01	0.002	
***Anaesthetist***						
*Anaesthetist 1*		Ref				
*Anaesthetist 2*		3.6	1.2	<0.01	0.000	
*Anaesthetist 5*		4.6	0.9	<0.01	0.001	
*Anaesthetist 9*		-3.4	1.1	<0.01	0.000	
*Anaesthetist 10*		3.5	0.9	<0.01	0.000	
***Session factors***						
***Session type***						
*AM list*		Ref				
***Theatre ***						
*Day Theatre 2*		Ref				
*Day Theatre 4*		2.4	0.8	<0.01	0.000	
*Day Theatre 5*		-1.5	0.4	<0.01	0.002	

**Table 3:** Multiple regression analysis model and list utilization in Main Theatres (only reference categories and retained model variables listed).

***Risk factor***	***Model coefficient***	***Beta coefficient***	***Beta Standard Error***	***p-value***	***Charge in R Square***	***Model R Square***
*Constant*	46.3	-	0.7	<0.01	-	0.621
***List volume (list-score units/hour)***		0.252	0.007	<0.01	0.334	
***List factors***						
***Adjusted overruns***						
*No overrun*		Ref				
*Overrun 1 (0–0.1)*		17.9	0.6	<0.01	0.051	
*Overrun 2 (0.11–0.2)*		25.2	0.8	<0.01	0.038	
*Overrun 3 (0.21–0.3)*		33.7	1.0	<0.01	0.038	
*Overrun 4 (>0.31)*		54.0	1.0	<0.01	0.118	
***Adjusted late-starts***						
*No late start*		Ref				
*Late-start 3 (0.21–0.3)*		-2.9	0.6	<0.01	0.001	
*Late-start 4 (>0.31)*		-12.0	0.9	<0.01	0.010	
***Session factors***						
***Session type***						
*AM list*		2.8	0.7	<0.01	0.001	
*All-day list*		Ref				
***Theatre***						
*MT2*		Ref				
*MT3*		2.8	0.9	<0.01	0.000	
*MT6*		3.1	0.6	<0.01	0.001	
*MT8*		7.7	3.3	0.02	0.000	
*MT10*		2.3	0.8	<0.01	0.000	
*Other 'theatres*		5.7	0.9	<0.01	0.000	
***Personnel factors***						
***Surgeon***						
*Surgeon 1*		Ref				
*Surgeon 2*		-4.3	1.4	<0.01	0.000	
*Surgeon 8*		-8.1	0.8	<0.01	0.007	
*Surgeon 9*		-4.4	2.1	0.03	0.000	
*Surgeon 10*		-4.1	1.2	<0.01	0.000	
*Surgeon 12*		-5.0	0.9	<0.01	0.000	
*Surgeon 13*		2.5	0.8	<0.01	0.001	
*Surgeon 14*		-4.4	1.3	<0.01	0.000	
*Surgeon 'Others'*		-8.2	0.6	<0.01	0.009	
***Anaesthetist***						
*Anaesthetist 1*		Ref				
*Anaesthetist 4*		-4.8	0.9	<0.01	0.007	
*Anaesthetist 5*		-4.4	1.7	<0.01	0.000	
*Anaesthetist 6*		3.2	0.9	<0.01	0.000	
*Anaesthetist 8*		-4.3	0.9	<0.01	0.003	
*Anaesthetist 10*		6.6	1.1	<0.01	0.000	
*Anaesthetist 14*		-7.9	1.6	<0.01	0.000	
*Anaesthetist 'Others'*		-1.5	0.5	<0.01	0.000	

The relative influence of the individual predictors within the model are summarised as the change in R-Square statistic. This statistic represents the impact that exclusion of the considered cofactor has upon the models overall explanatory capability. In both the day surgery and main theatre models the principal determinants of theatre list utilization were: the size of the operating list (p < 0.01) and whether or not the list overran (p < 0.01). Specifically, in the DS department the change in R Square statistic associated with 'operative list size' and overruns were 0.394 and 0.152 respectively. Other DS model cofactors including; late starts > 1 hour (p < 0.01, change in R Square statistic = 0.014), as well as individual surgeons and anaesthetists demonstrated a significant, but small, independent influence on the models explanatory power (see Table 2). In the DS model, session type demonstrated no independent relationship with utilization rates once adjusted for other factors. Similarly, only two of the five theatre suites used for surgery in the day unit demonstrated a small independent influence on utilization (see Table 2). In the MT model, operating list size (p < 0.01, change in R Square statistic = 0.334) and overrun categories (p < 0.01, change in R Square statistic 0.038 – 0.118 for categories 'Overrun 1–4') demonstrated the greatest independent influence on list utilization rates of all model covariates (see Table 3). By comparison, the relative influence of other covariates, including session type, individual theatre suites, as well as specific surgical and anaesthetic practitioners, was significant but modest (see Table 3).

## Discussion

Theatre utilization represents a qualitative measure of theatre time usage. Since the publication of the 'STEP Guide to Improving Operating Theatre Performance' by the Modernisation Agency [8] and two national Audit Commission reports on Operating Theatres [6,7], utilization has become the principal managerial measure of theatre performance across Trusts in the United Kingdom. Little investigation has however hitherto been conducted to determine the validity of theatre utilization, as a marker of theatre performance, in the public sector setting.

The results of this study pertain to a single centre. Direct extrapolation of the study results to other Trusts, or even other specialties, is not possible. Many problems within NHS hospitals are however shared between centres. Although only data from one centre was used, the results and conclusions of this study are therefore, by proxy, of relevance to other units. The principal driver of theatre list utilization within this study was operating list size in both the DS and MT departments. In reality, the size of operating lists is often determined by the availability of resources such as ward or high dependency beds. MT utilization in a public sector hospital is therefore possibly determined largely by bed capacity. Importantly, this cannot be directly substantiated in the current study as bed capacity data was not a collected variable. If however a relationship between bed capacity and main theatre utilization is accepted – then, in the context of declining numbers of ward beds in NHS hospitals [17], utilization of MT units may decline also. In the DS department low operating list volumes frequently arise due to late 'patient' or 'hospital' cancellations. As such, low theatre utilization rates in this context may require specific corrective measures to ensure that all list patients attend, and are fit, for their operations. To this end the Modernisation Agency has issued specific practical advice on administrative and clinical measures aimed at reducing cancelled operations [18]. Despite this, using measures of surgical workload to measure: intended admissions, patient cancellations and eventual operative list volume might represent more useful managerial data than theatre utilization rates.

In our study a strong association between theatre utilization and list overruns was observed in both the DS and MT departments. This is understandable as, 'allocated session time' was used to calculate list utilization rates. The rationale for not adjusting the session time to include overtime is that it is not current standard managerial practice to do so in NHS hospitals. Although dangers can arise from the extrapolation of the findings of a single centre study the relationship demonstrated here between utilization and overruns is logical when the basis of the equation used to calculate utilization is considered. Hence, overrunning lists probably serve to inflate utilization rates reported by NHS Trusts. List overruns are however a significant source of inefficiency in NHS theatres as they are costly in terms of overtime payments and staff morale. Confusingly therefore, Trusts where theatre overruns occur commonly are likely to report high utilization rates also. Although there is evidence that some researchers investigating theatre time usage have adjusted utilization methodology to account for overtime [19] there is little evidence that this is being performed in NHS Trusts. In fact, the inclusion of units with reported utilization rates in excess of 100% in the latest Audit Commission report suggests that adjustment was not made by at least some centres.

In various units individualised theatre utilization rates are routinely sent to surgical and anaesthetic staff as a marker of service performance. The results of this study question the validity of this exercise. Specifically, surgeons displayed significant independent differences in the determination of list utilization in both the DS and MT settings where coefficients ranged from 3.8 to -13.2% and 0 to -8.2% between them in these differing contexts respectively. Although differences between individuals were significant their overall influence on utilization was modest compared to that of operative volume and whether, or not, list overruns occurred. As such, unadjusted individualised utilization rates are more likely to represent the influence of the latter factors rather than the specific performance of theatre personnel. For this reason the use of unadjusted utilization rates could be misrepresentative if used for service activity monitoring of surgical personnel.

For the reasons cited above an optimal level of theatre utilization that is appropriate to NHS theatres is difficult to define. The Audit Commission reported that the average Trust utilizes 73% of their total planned session time but theatre utilization across Trusts varied between 41 per cent and 103 per cent. These figures compare broadly to estimates of utilization detected in other investigations into theatre time usage across a variety of specialties in the United Kingdom [19-22]. The Audit Commission methodology incorporates however the attrition of theatre time brought about by cancelled operating lists and national estimates suggest that these comprise approximately 10% of all planned Trust sessions [23]. As such, utilization rates that do not account for cancelled sessions will overestimate utilization. The extent to which this methodology has previously been applied by Trusts is uncertain. In the short-term however, the Toolkit devised by the Modernisation Agency [8] should facilitate standardisation of theatre utilization calculation. Overall, the Audit Commission suggests an optimal 'end utilization' performance target of 77% [6]. Presently however, this study suggests that – whilst unaccounted discrepancies exist between Trusts': overrun rates, inpatient bed facilities and their methodology used to calculate utilization – some scepticism regarding the validity of a 'target' utilization rate for NHS theatres should be maintained. In the future, quantitative measures of surgical service workload, such as Human Resource Group (HRG) tariffs, are likely to predominate over theatre utilization. Definition of an actual service 'output' in NHS Trusts has facilitated political, strategic as well as operational decision-making. A possible extension of this to the operating theatre environment may be to use 'HRG output per theatre per time-period' as an efficiency measure. Irrespective however of the validity of a specific tool that quantifies theatre effectiveness; improving elective theatre efficiency demands a broad perspective over the entire surgical pathway.

## Conclusion

Maximising theatre usage is obviously desirable in the NHS. Variation between Trusts, in terms of overrun rates and inconsistent methodologies used to calculate utilization, impede its meaningful use as a tool that can benchmark theatre performance. Extreme utilization rates do however merit managerial investigation. Quantitative measures of theatre workload and efficiency are likely to be used for decision-making in the future.

## Competing interests

In 2003 OF received a £30,000 contribution towards salary from the South East London Strategic Health Authority whilst undertaking a period of research.

## Authors' contributions

OF contributed to study design, data acquisition, statistical data analysis and drafting of the manuscript. PT contributed to the multivariate statistics and data analysis as well as drafting of the manuscript. AMcG participated in study design, results appraisal and contributed towards the intellectual content of the discussion. SP and JR both were responsible for critical appraisal of the results as well as offering a contribution towards the intellectual content of the discussion. AL co-conceived the study and shared responsibility for its design. He also co-ordinated as well as critically appraised the results and helped with drafting of the manuscript. All authors read and approved the final manuscript.

## Pre-publication history

The pre-publication history for this paper can be accessed here:


